# Supplementary material for: Targeted and Checkpoint Inhibitor Therapy of Metastatic Malignant Melanoma in Germany, 2000–2016
Source: Cancers (Basel). 2020 Aug 20;12(9):2354. doi: 10.3390/cancers12092354 (PMC7565277; doi:10.3390/cancers12092354)
Supplement: Supplementary file 1 [file cancers-12-02354-s001.pdf]

Table S1: Complete Cox regression results for survival since diagnosis of first metastasis, adjusted for age at metastasis, sex, region, anatomic site of the primary tumor, melanoma subtype of the primary tumor, metastasis type, and therapy (*n* = 3871; events: 2965).

	**β**	**se(β)**	**Hazard ratio (95%-CI)**	**z**	**p**
Age at metastasis	0.011	0.001	1.011 (1.009; 1.014)	8.011	< 0.001
Sex: female (ref: male)	-0.140	0.039	0.869 (0.806; 0.938)	-3.619	< 0.001
Region: Eastern Germany (ref: Western Germany)	0.385	0.045	1.470 (1.347; 1.604)	8.653	< 0.001
Anatomic site of primary tumor (localization) (reference: unspecified/overlapping)					
Head/neck	-0.079	0.085	0.924 (0.782; 1.093)	-0.924	0.355
Interaction Head/neck with time since metastasis	0.243	0.059	1.275 (1.137; 1.430)	4.149	< 0.001
Trunk	-0.112	0.079	0.894 (0.766; 1.043)	-1.423	0.155
Interaction Trunk with time since metastasis	0.115	0.052	1.122 (1.014; 1.242)	2.237	0.025
Extremities	-0.152	0.076	0.859 (0.740; 0.998)	-1.993	0.046
Interaction Extremities with time since metastasis	0.211	0.051	1.235 (1.118; 1.365)	4.135	< 0.001
Melanoma subtype of primary tumor (morphology) (reference: other)					
Malignant melanoma, NOS	-0.101	0.086	0.904 (0.764; 1.069)	-1.182	0.237
Nodular melanoma	0.117	0.088	1.124 (0.946; 1.336)	1.334	0.182
Lentigo maligna melanoma	-0.160	0.140	0.852 (0.647; 1.122)	-1.141	0.254
Superficial spreading melanoma	-0.011	0.093	0.989 (0.824; 1.188)	-0.115	0.908
Acral lentiginous melanoma	0.011	0.120	1.011 (0.799; 1.280)	0.090	0.928
Metachronous metastasis (ref: no metachronous metastasis)	0.200	0.048	1.222 (1.113; 1.341)	4.217	< 0.001
Interaction metachronous metastasis with tsm	-0.162	0.034	0.851 (0.795; 0.910)	-4.704	< 0.001
Chemotherapy: yes (ref: no)	0.212	0.063	1.237 (1.094; 1.398)	3.387	< 0.001
Interaction Chemotherapy: yes with tsm	0.236	0.051	1.266 (1.146; 1.398)	4.632	< 0.001
Interferon therapy: yes (ref: no)	-0.107	0.052	0.899 (0.812; 0.995)	-2.061	0.039
Interaction Interferon therapy: yes with tsm	0.220	0.039	1.246 (1.154; 1.346)	5.623	< 0.001
Immune checkpoint inhibitor therapy: yes (ref: no)	-0.020	0.145	0.980 (0.738; 1.301)	-0.139	0.889
Interaction Immune checkpoint inhibitor therapy: yes with tsm	0.310	0.125	1.363 (1.067; 1.742)	2.474	0.013
Targeted therapy: yes (ref: no)	-0.185	0.067	0.831 (0.729; 0.948)	-2.752	0.006
Interaction Targeted therapy: yes with tsm	0.461	0.059	1.585 (1.412; 1.779)	7.799	< 0.001

NOS = Not otherwise specified, tsm = time since metastasis

Table S2: Cox regression results for five subsets with different cutoff values per registry & year for shares of patients for whom no therapy was documented.

**Cutoff value: maximum share of no documented therapies per registry & year (%)**	**50**	**67**	**75**	**80**	**90**
n (share of included patients)	1053 (27%)	1970 (51%)	2642 (68%)	3039 (79%)	3619 (94%)
	**HR (95%-CI)**	**HR (95%-CI)**	**HR (95%-CI)**	**HR (95%-CI)**	**HR (95%-CI)**
Age at metastasis	1.018 (1.012; 1.024)	1.013 (1.009; 1.017)	1.011 (1.007; 1.014)	1.010 (1.007; 1.013)	1.011 (1.008; 1.014)
Sex: female (ref: male)	0.924 (0.791; 1.080)	0.870 (0.781; 0.970)	0.884 (0.806; 0.970)	0.895 (0.822; 0.976)	0.870 (0.804; 0.941)
Region: Eastern Germany (ref: Western Germany)	1.739 (1.375; 2.200)	1.535 (1.331; 1.771)	1.559 (1.387; 1.751)	1.543 (1.384; 1.719)	1.537 (1.398; 1.691)
Anatomic site of primary tumor (localization) (reference: unspecified/overlapping)					
Head/neck	1.061 (0.744; 1.513)	0.946 (0.743; 1.204)	0.947 (0.764; 1.175)	0.951 (0.775; 1.168)	0.924 (0.782; 1.093)
Interaction Head/neck with time since metastasis	1.134 (0.870; 1.479)	1.283 (1.069; 1.539)	1.286 (1.099; 1.504)	1.287 (1.114; 1.487)	1.275 (1.137; 1.430)
Trunk	1.000 (0.713; 1.401)	0.934 (0.742; 1.175)	0.924 (0.755; 1.131)	0.930 (0.767; 1.128)	0.894 (0.766; 1.043)
Interaction Trunk with time since metastasis	0.981 (0.765; 1.257)	1.045 (0.886; 1.232)	1.096 (0.952; 1.261)	1.140 (1.000; 1.299)	1.122 (1.014; 1.242)
Extremities	0.893 (0.645; 1.237)	0.814 (0.653; 1.015)	0.825 (0.678; 1.004)	0.834 (0.690; 1.007)	0.859 (0.740; 0.998)
Interaction Extremities with time since metastasis	1.078 (0.843; 1.378)	1.126 (0.958; 1.323)	1.179 (1.026; 1.355)	1.212 (1.065; 1.379)	1.235 (1.118; 1.365)
Melanoma subtype of primary tumor (morphology) (reference: other)					
Malignant melanoma, NOS	0.966 (0.652; 1.430)	0.884 (0.691; 1.132)	0.892 (0.723; 1.101)	0.893 (0.738; 1.080)	0.904 (0.764; 1.069)
Nodular melanoma	1.033 (0.686; 1.556)	1.078 (0.836; 1.389)	1.086 (0.876; 1.347)	1.087 (0.896; 1.319)	1.124 (0.946; 1.336)
Lentigo maligna melanoma	1.054 (0.571; 1.946)	0.826 (0.538; 1.269)	0.818 (0.578; 1.157)	0.811 (0.593; 1.110)	0.852 (0.647; 1.122)
Superficial spreading melanoma	1.209 (0.789; 1.853)	0.998 (0.764; 1.304)	0.975 (0.778; 1.224)	0.980 (0.799; 1.202)	0.989 (0.824; 1.188)
Acral lentiginous melanoma	1.353 (0.812; 2.254)	0.998 (0.708; 1.405)	0.972 (0.727; 1.301)	0.960 (0.740; 1.247)	1.011 (0.799; 1.280)
Metachronous metastasis (ref: no metachronous metastasis)	1.187 (0.984; 1.432)	1.205 (1.054; 1.378)	1.204 (1.073; 1.351)	1.187 (1.066; 1.321)	1.214 (1.102; 1.337)
Interaction metachronous metastasis with tsm	0.813 (0.703; 0.940)	0.827 (0.745; 0.917)	0.871 (0.798; 0.951)	0.880 (0.812; 0.953)	0.880 (0.820; 0.945)
Chemotherapy: yes (ref: no)	1.436 (1.148; 1.796)	1.223 (1.048; 1.428)	1.262 (1.099; 1.450)	1.282 (1.124; 1.461)	1.233 (1.089; 1.395)
Interaction Chemotherapy: yes with tsm	1.491 (1.224; 1.818)	1.370 (1.199; 1.566)	1.322 (1.176; 1.486)	1.292 (1.158; 1.440)	1.254 (1.134; 1.387)

Interferon therapy: yes (ref: no)	0.971 (0.790; 1.193)	0.875 (0.765; 1.001)	0.908 (0.810; 1.018)	0.871 (0.782; 0.970)	0.895 (0.808; 0.992)
Interaction Interferon therapy: yes with tsm	1.277 (1.090; 1.496)	1.224 (1.106; 1.355)	1.243 (1.138; 1.358)	1.208 (1.113; 1.311)	1.212 (1.121; 1.311)
Immune checkpoint inhibitor therapy: yes (ref: no)	1.298 (0.890; 1.893)	1.027 (0.738; 1.428)	1.049 (0.776; 1.420)	0.991 (0.736; 1.335)	0.964 (0.721; 1.288)
Interaction Immune checkpoint inhibitor therapy: yes with tsm	1.246 (0.904; 1.718)	1.273 (0.957; 1.695)	1.391 (1.062; 1.821)	1.390 (1.066; 1.811)	1.331 (1.038; 1.708)
Targeted therapy: yes (ref: no)	1.010 (0.831; 1.227)	0.872 (0.749; 1.016)	0.849 (0.737; 0.979)	0.835 (0.727; 0.959)	0.829 (0.726; 0.947)
Interaction Targeted therapy: yes with tsm	1.628 (1.379; 1.920)	1.579 (1.380; 1.806)	1.546 (1.363; 1.754)	1.512 (1.339; 1.708)	1.546 (1.376; 1.738)

n = number of observations, NOS = Not otherwise specified, tsm = time since metastasis

Table S3: Cox regression results for patients with an overall survival of at least 31 days since metastasis (n = 3634, events: 2748).

	**Hazard ratio (95%-CI)**
Age at metastasis	1.010 (1.007; 1.013)
Sex: female (ref: male)	0.880 (0.814; 0.952)
Region: Eastern Germany (ref: Western Germany)	1.498 (1.368; 1.640)
Anatomic site of primary tumor (localization) (reference: unspecified/overlapping)	
Head/neck	0.918 (0.776; 1.087)
Interaction Head/neck with time since metastasis	1.321 (1.141; 1.529)
Trunk	0.887 (0.759; 1.037)
Interaction Trunk with time since metastasis	1.154 (1.013; 1.315)
Extremities	0.851 (0.732; 0.990)
Interaction Extremities with time since metastasis	1.283 (1.129; 1.458)
Melanoma subtype of primary tumor (morphology) (reference: other)	
Malignant melanoma, NOS	0.897 (0.753; 1.067)
Nodular melanoma	1.102 (0.922; 1.318)
Lentigo maligna melanoma	0.845 (0.634; 1.126)
Superficial spreading melanoma	0.976 (0.808; 1.180)
Acral lentiginous melanoma	1.039 (0.816; 1.324)
Metachronous metastasis (ref: no metachronous metastasis)	1.240 (1.128; 1.362)
Interaction metachronous metastasis with tsm	0.845 (0.779; 0.918)
Chemotherapy: yes (ref: no)	1.261 (1.115; 1.427)
Interaction Chemotherapy: yes with tsm	1.207 (1.078; 1.352)
Interferon therapy: yes (ref: no)	0.896 (0.809; 0.992)
Interaction Interferon therapy: yes with tsm	1.218 (1.111; 1.336)
Immune checkpoint inhibitor therapy: yes (ref: no)	0.983 (0.739; 1.307)
Interaction Immune checkpoint inhibitor therapy: yes with tsm	1.332 (1.009; 1.758)
Targeted therapy: yes (ref: no)	0.833 (0.729; 0.952)
Interaction Targeted therapy: yes with tsm	1.552 (1.366; 1.763)

NOS = Not otherwise specified, tsm = time since metastasis

Table S4: Cox regression results for patients from 2012 and thereafter only and with patients treated with ICI before 2015 excluded (*n* = 1502, events: 941).

	**Hazard ratio (95%-CI)**
Age at metastasis	1.015 (1.009; 1.020)
Sex: female (ref: male)	0.913 (0.798; 1.044)
Region: Eastern Germany (ref: Western Germany)	1.345 (1.149; 1.575)
Anatomic site of primary tumor (localization) (reference: unspecified/overlapping)	
Head/neck	0.989 (0.694; 1.411)
Interaction Head/neck with time since metastasis	1.309 (1.042; 1.644)
Trunk	0.947 (0.678; 1.322)
Interaction Trunk with time since metastasis	1.073 (0.877; 1.312)
Extremities	0.929 (0.670; 1.288)
Interaction Extremities with time since metastasis	1.290 (1.054; 1.580)
Melanoma subtype of primary tumor (morphology) (reference: other)	
Malignant melanoma, NOS	0.844 (0.639; 1.114
Nodular melanoma	1.040 (0.779; 1.389)
Lentigo maligna melanoma	0.727 (0.460; 1.151)
Superficial spreading melanoma	1.032 (0.759; 1.404)
Acral lentiginous melanoma	0.957 (0.627; 1.461)
Metachronous metastasis (ref: no metachronous metastasis)	1.021 (0.836; 1.247)
Interaction metachronous metastasis with tsm	0.972 (0.844; 1.120)
Chemotherapy: yes (ref: no)	1.317 (0.915; 1.893)
Interaction Chemotherapy: yes with tsm	1.264 (0.938; 1.703)
Interferon therapy: yes (ref: no)	1.018 (0.831; 1.246)
Interaction Interferon therapy: yes with tsm	1.362 (1.160; 1.599)
Immune checkpoint inhibitor therapy: yes (ref: no)	1.639 (0.980; 2.741)
Interaction Immune checkpoint inhibitor therapy: yes with tsm	1.833 (1.180; 2.849)
Targeted therapy: yes (ref: no)	1.192 (0.967; 1.469)
Interaction Targeted therapy: yes with tsm	1.766 (1.454; 2.145)

NOS = Not otherwise specified, tsm = time since metastasis

Table S5: Cox regression results including an additional variable indicating whether primary diagnosis was made since July 2008 (*n* = 3871, events: 2965).

	**Hazard ratio (95%-CI)**
Age at metastasis	1.011 (1.008; 1.014)
Sex: female (ref: male)	0.870 (0.806; 0.938)
Region: Eastern Germany (ref: Western Germany)	1.474 (1.350; 1.608)
Anatomic site of primary tumor (localization) (reference: unspecified/overlapping)	
Head/neck	0.920 (0.778; 1.088)
Interaction Head/neck with time since metastasis	1.274 (1.136; 1.429)
Trunk	0.892 (0.765; 1.041)
Interaction Trunk with time since metastasis	1.122 (1.014; 1.241)
Extremities	0.858 (0.739; 0.996)
Interaction Extremities with time since metastasis	1.234 (1.117; 1.364)
Melanoma subtype of primary tumor (morphology) (reference: other)	
Malignant melanoma, NOS	0.904 (0.765; 1.070)
Nodular melanoma	1.125 (0.947; 1.336)
Lentigo maligna melanoma	0.857 (0.651; 1.129)
Superficial spreading melanoma	0.995 (0.829; 1.195)
Acral lentiginous melanoma	1.011 (0.799; 1.279)
Metachronous metastasis (ref: no metachronous metastasis)	1.227 (1.118; 1.347)
Interaction metachronous metastasis with tsm	0.852 (0.797; 0.912)
Chemotherapy: yes (ref: no)	1.243 (1.099; 1.406)
Interaction Chemotherapy: yes with tsm	1.266 (1.146; 1.399)
Interferon therapy: yes (ref: no)	0.897 (0.810; 0.992)
Interaction Interferon therapy: yes with tsm	1.245 (1.153; 1.345)
Immune checkpoint inhibitor therapy: yes (ref: no)	0.958 (0.720; 1.274)
Interaction Immune checkpoint inhibitor therapy: yes with tsm	1.370 (1.071; 1.752)
Targeted therapy: yes (ref: no)	0.821 (0.719; 0.938)
Interaction Targeted therapy: yes with tsm	1.589 (1.415; 1.784)
Primary diagnosis as from July 2008: yes (ref: no)	1.055 (0.978; 1.139)

NOS = Not otherwise specified, tsm = time since metastasis

Table S6: Cox regression results for the overall sample and for the subgroups of patients from Eastern Germany (n = 2791, events: 2231) and patients from Western Germany (n = 1080, events: 734).

		Strata: Region	
	**Overall sample Hazard ratio (95%-CI)**	**Eastern Germany Hazard ratio (95%-CI)**	**Western Germany Hazard ratio (95%-CI)**
Age at metastasis	1.011 (1.009; 1.014)	1.011 (1.008; 1.015)	1.012 (1.006; 1.017)
Sex: female (ref: male)	0.869 (0.806; 0.938)	0.855 (0.783; 0.933)	0.939 (0.807; 1.093)
Region: Eastern Germany (ref: Western Germany)	1.470 (1.347; 1.604)	-	-
Anatomic site of primary tumor (localization) (reference: unspecified/overlapping)			
Head/neck	0.924 (0.782; 1.093)	0.986 (0.794; 1.225)	0.823 (0.620; 1.093)
Interaction Head/neck with time since metastasis	1.275 (1.137; 1.430)	1.076 (0.921; 1.257)	1.574 (1.302; 1.903)
Trunk	0.894 (0.766; 1.043)	0.966 (0.791; 1.181)	0.735 (0.567; 0.953)
Interaction Trunk with time since metastasis	1.122 (1.014; 1.242)	0.953 (0.828; 1.098)	1.313 (1.127; 1.530)
Extremities	0.859 (0.740; 0.998)	0.891 (0.732; 1.083)	0.815 (0.639; 1.040)
Interaction Extremities with time since metastasis	1.235 (1.118; 1.365)	1.038 (0.903; 1.194)	1.486 (1.278; 1.728)
Melanoma subtype of primary tumor (morphology) (reference: other)			
Malignant melanoma, NOS	0.904 (0.764; 1.069)	0.886 (0.716; 1.098)	0.935 (0.709; 1.233)
Nodular melanoma	1.124 (0.946; 1.336)	1.063 (0.858; 1.317)	1.329 (0.980; 1.802)
Lentigo maligna melanoma	0.852 (0.647; 1.122)	0.839 (0.611; 1.152)	0.796 (0.429; 1.475)
Superficial spreading melanoma	0.989 (0.824; 1.188)	0.927 (0.738; 1.166)	1.149 (0.843; 1.567)
Acral lentiginous melanoma	1.011 (0.799; 1.280)	0.981 (0.738; 1.305)	1.110 (0.720; 1.711)
Metachronous metastasis (ref: no metachronous metastasis)	1.222 (1.113; 1.341)	1.223 (1.095; 1.366)	1.223 (1.019; 1.467)
Interaction metachronous metastasis with tsm	0.851 (0.795; 0.910)	0.910 (0.841; 0.985)	0.709 (0.623; 0.806)
Chemotherapy: yes (ref: no)	1.237 (1.094; 1.398)	1.175 (1.013; 1.362)	1.348 (1.057; 1.719)
Interaction Chemotherapy: yes with tsm	1.266 (1.146; 1.398)	1.215 (1.082; 1.365)	1.340 (1.084; 1.657)
Interferon therapy: yes (ref: no)	0.899 (0.812; 0.995)	0.919 (0.816; 1.034)	0.831 (0.669; 1.033)
Interaction Interferon therapy: yes with tsm	1.246 (1.154; 1.346)	1.252 (1.146; 1.369)	1.239 (1.044; 1.470)

Immune checkpoint inhibitor therapy: yes (ref: no)	0.980 (0.738; 1.301)	0.914 (0.601; 1.388)	0.950 (0.625; 1.443)
Interaction Immune checkpoint inhibitor therapy: yes with tsm	1.363 (1.067; 1.742)	1.200 (0.861; 1.674)	1.502 (1.007; 2.239)
Targeted therapy: yes (ref: no)	0.831 (0.729; 0.948)	0.837 (0.722; 0.971)	0.807 (0.571; 1.141)
Interaction Targeted therapy: yes with tsm	1.585 (1.412; 1.779)	1.614 (1.417; 1.839)	1.378 (1.021; 1.859)

NOS = Not otherwise specified, tsm = time since metastasis

Table S7: Cox regression results for the overall sample and for the subgroups of patients with synchronous metastasis (*n* = 1352, events: 1029) and patients with metachronous metastasis (*n* = 2519, events: 1936).

		**Strata: Type of metastasis**	
	**Overall sample**	**Synchronous metastasis**	**Metachronous metastasis**
	**Hazard ratio (95%-CI)**	**Hazard ratio (95%-CI)**	**Hazard ratio (95%-CI)**
Age at metastasis	1.011 (1.009; 1.014)	1.014 (1.009; 1.018)	1.011 (1.007; 1.014)
Sex: female (ref: male)	0.869 (0.806; 0.938)	0.795 (0.699; 0.905)	0.903 (0.822; 0.992)
Region: Eastern Germany (ref: Western Germany)	1.470 (1.347; 1.604)	1.504 (1.318; 1.716)	1.423 (1.264; 1.603)
Anatomic site of primary tumor (localization) (reference: unspecified/overlapping)			
Head/neck	0.924 (0.782; 1.093)	0.954 (0.769; 1.183)	1.872 (1.095; 3.200)
Interaction Head/neck with time since metastasis	1.275 (1.137; 1.430)	1.278 (1.093; 1.493)	1.348 (0.992; 1.832)
Trunk	0.894 (0.766; 1.043)	0.817 (0.677; 0.985)	1.911 (1.125; 3.246)
Interaction Trunk with time since metastasis	1.122 (1.014; 1.242)	1.135 (1.001; 1.287)	1.194 (0.886; 1.611)
Extremities	0.859 (0.740; 0.998)	0.767 (0.640; 0.918)	1.881 (1.111; 3.183)
Interaction Extremities with time since metastasis	1.235 (1.118; 1.365)	1.290 (1.138; 1.462)	1.319 (0.978; 1.777)
Melanoma subtype of primary tumor (morphology) (reference: other)			
Malignant melanoma, NOS	0.904 (0.764; 1.069)	0.781 (0.617; 0.990)	1.027 (0.808; 1.305)
Nodular melanoma	1.124 (0.946; 1.336)	1.021 (0.775; 1.345)	1.251 (0.989; 1.581)
Lentigo maligna melanoma	0.852 (0.647; 1.122)	0.786 (0.440; 1.402)	0.995 (0.713; 1.389)
Superficial spreading melanoma	0.989 (0.824; 1.188)	0.782 (0.574; 1.064)	1.154 (0.904; 1.474)
Acral lentiginous melanoma	1.011 (0.799; 1.280)	0.971 (0.609; 1.550)	1.116 (0.831; 1.498)
Metachronous metastasis (ref: no metachronous metastasis)	1.222 (1.113; 1.341)	-	-
Interaction metachronous metastasis with tsm	0.851 (0.795; 0.910)	-	-
Chemotherapy: yes (ref: no)	1.237 (1.094; 1.398)	1.397 (1.193; 1.636)	1.093 (0.883; 1.352)
Interaction Chemotherapy: yes with tsm	1.266 (1.146; 1.398)	1.353 (1.189; 1.540)	1.175 (0.997; 1.386)
Interferon therapy: yes (ref: no)	0.899 (0.812; 0.995)	0.477 (0.353; 0.644)	1.042 (0.926; 1.172)
Interaction Interferon therapy: yes with tsm	1.246 (1.154; 1.346)	1.276 (1.005; 1.618)	1.327 (1.213; 1.452)

Immune checkpoint inhibitor therapy: yes (ref: no)	0.980 (0.738; 1.301)	0.876 (0.645; 1.191)	2.619 (1.137; 6.030)
Interaction Immune checkpoint inhibitor therapy: yes with tsm	1.363 (1.067; 1.742)	1.354 (1.043; 1.757)	2.057 (0.864; 4.898)
Targeted therapy: yes (ref: no)	0.831 (0.729; 0.948)	0.715 (0.557; 0.917)	0.918 (0.772; 1.091)
Interaction Targeted therapy: yes with tsm	1.585 (1.412; 1.779)	1.796 (1.455; 2.216)	1.569 (1.348; 1.827)

NOS = Not otherwise specified, tsm = time since metastasis
